# In Vitro Antibacterial Interaction of Doripenem and Amikacin against Multidrug-Resistant *Acinetobacter baumannii*, *Pseudomonas aeruginosa*, and *Klebsiella pneumoniae* Isolates

**DOI:** 10.1155/2018/1047670

**Published:** 2018-07-03

**Authors:** Tonny Loho, Ninik Sukartini, Dalima A. W. Astrawinata, Suzanna Immanuel, Diana Aulia, Ika Priatni

**Affiliations:** Infectious Diseases Division, Department of Clinical Pathology, Faculty of Medicine, Universitas Indonesia, Jakarta, Indonesia

## Abstract

Evaluation of the in vitro interaction of doripenem and amikacin against *Acinetobacter baumannii*, *Pseudomonas aeruginosa*, and *Klebsiella pneumoniae* was done by classifying them into four groups: doripenem and amikacin sensitive (DOR-S/AMK-S), doripenem sensitive and amikacin resistant (DOR-S/AMK-R), doripenem resistant and amikacin sensitive (DOR-R/AMK-S), and both doripenem and amikacin resistant (DOR-R/AMK-R). The MIC of each antibiotic and their combination was obtained using the Etest method. The fractional inhibitory concentration index was calculated to classify the results as synergistic, additive, indifferent, or antagonistic interaction. In the DOR-S/AMK-S class, 1 isolate of *A. baumannii* showed synergy and the other 5 showed additive results, 5 isolates of *P. aeruginosa* showed additive and 1 isolate showed indifferent result, and 2 isolates of *K. pneumoniae* showed additive and the other 4 showed indifferent results. In the DOR-S/AMK-R class, 3 isolates of *A. baumannii* showed additive and the other 3 showed indifferent results, 2 isolates of *P. aeruginosa* showed indifferent results, and 1 isolate of *K. pneumoniae* showed additive and the other 5 showed indifferent results. In the DOR-R/AMK-S class, 1 isolate of *A. baumannii* showed additive and the other 5 showed indifferent results, 1 isolate of *P. aeruginosa* showed additive and the other 5 showed indifferent results, and 4 isolates of *K. pneumoniae* showed additive and the other 2 showed indifferent results. In the DOR-R/AMK-R class, 6 isolates of *A. baumannii* showed indifferent results, 1 isolate of *P. aeruginosa* showed additive and the other 5 showed indifferent results, and 1 isolate of *K. pneumoniae* showed additive and the other 5 showed indifferent results. Synergy occurred in only 1 (1.5%) isolate. Additive interaction occurred in 24 (35.3%) isolates, and indifferent interaction occurred in 43 (63.2%) isolates. Doripenem sensitive combined with amikacin sensitive reduced MIC significantly in all bacterial isolates when compared to single MIC of each antibiotic.

## 1. Background

The wide overuse or misuse of antibiotics has been followed by the emergence of resistant bacteria causing healthcare-associated and community-acquired infections worldwide [1]. Many of the pathogens related to the epidemic infectious diseases in humans have evolved into multidrug-resistant (MDR) bacteria [2]. Some isolates change further and become extended drug-resistant (XDR) or pandrug-resistant (PDR) pathogens. Antibiotic resistance pattern in Cipto Mangunkusumo Hospital (CMH), Jakarta, showed that the most prevalent PDR bacteria last year were *Acinetobacter baumannii*, *Klebsiella pneumoniae*, and *Pseudomonas aeruginosa* [3, 4].

Empirical treatment with antibiotics combination has been recommended in severe sepsis and septic shock to reduce mortality such as meropenem and amikacin, meropenem and levofloxacin, and carbapenem (meropenem, imipenem, and doripenem) and polymyxin [5, 6]. In vitro studies showed that combining antimicrobial agents could be more effective against resistant pathogens than the single antibiotic. It can be used to evaluate the efficacy of an antibiotic combination to treat severe infection caused by MDR Gram-negative bacteria [7]. A study reported that the combination of carbapenem and polymyxin resulted in high synergy against *Acinetobacter baumannii*, among which doripenem was superior to meropenem and imipenem [5]. Other study found that the combination of cefepime and amikacin or meropenem and amikacin resulted in high synergy against MDR *P. aeruginosa* isolates from intensive care unit patients [8]. Higher synergy can be achieved by combining 2 or 3 antibiotics of aminoglycosides, aztreonam, carbapenem, colistin, rifampin, tigecycline, and fosfomycin [7].

Doripenem is a relatively new carbapenem and has not been widely studied as meropenem [9]. Therefore, it is interesting to know how doripenem will interact with other class of antibiotic in combination. Aminoglycosides has been a preferable agent for combining antibiotics due to its wide spectrum and good interaction with antibiotics that act on the bacterial cell wall, such as penicillin, cephalosporins, monobactam, carbapenems, and glycopeptides [6, 7]. This study was aimed to evaluate the interaction of doripenem and amikacin combination against three resistant Gram-negative bacteria, that is, *Acinetobacter baumannii*, *Klebsiella pneumoniae*, and *Pseudomonas aeruginosa*.

## 2. Methods

### 2.1. Study Design and Specimens

This study was a laboratory experimental test in the Department of Clinical Pathology, Cipto Mangunkusumo National Central General Hospital (CMNCGH), Jakarta, between April and October 2016. Study materials were bacterial isolates of *Acinetobacter baumannii*, *Pseudomonas aeruginosa*, and *Klebsiella pneumoniae*. Specimens were collected from hospitalized or ambulatory patients in CMNCGH, which were sent to the Microbiology Laboratory of Clinical Pathology Department.

### 2.2. Bacterial Identification and Antibiotic Susceptibility Test

Bacteria were identified by using colony morphology identification, Gram staining, and biochemical testing. Isolates were included if the antibiotic susceptibility testing using Kirby–Bauer disk diffusion method showed resistant or sensitive to doripenem and amikacin. Isolates with intermediate resistant were excluded from analyses. The quality of susceptibility testing was controlled by using *Escherichia coli* ATCC 25922 against Enterobacteriaceae and *Pseudomonas aeruginosa* ATCC 27853 against nonfermenter Gram-negative rod as recommended by the Clinical and Laboratory Standards Institute (CLSI) guidelines [10]. When the results were within the allowed concentration range, the same isolates underwent a second antibiotic susceptibility test using Kirby–Bauer disk difussion method to confirm their susceptibility against doripenem and amikacin. Antibiotic interaction test was done only if the second antibiotic susceptibility test confirmed the results of the first test. Resistance was defined based on the inhibitory zone diameter on the diffusion disk method by CLSI 2016. Resistant bacteria were grouped as MDR, if nonsusceptible to ≥1 agent in ≥3 antimicrobial categories; XDR, if nonsusceptible to ≥1 agent in all but ≤2 categories; PDR, if nonsusceptible to all antimicrobial agents listed [11].

### 2.3. In Vitro Antibiotic Interaction Test

Antibiotic interaction test was done using the Etest method (Liofilchem® MIC Test Strip, Italy) of doripenem and amikacin on each bacterial isolates. Strips of both antibiotics were placed on the surface of preinoculated Mueller-Hinton (MH) agar with the scale upside. Strips were gently pressed on the agar surface and were left there for 15 minutes. Then, the strips and agar were incubated for 18–24 h at 35°C. The minimal inhibitory concentration (MIC) of each antibiotic was the value at which the inhibition zone intersected the scale on E strip [12]. The concentration range was 0.002–32 *µ*g/mL for doripenem (MIC_DOR_) and 0.016–256 *µ*g/mL for amikacin (MIC_AMK_).

**Figure 1:**
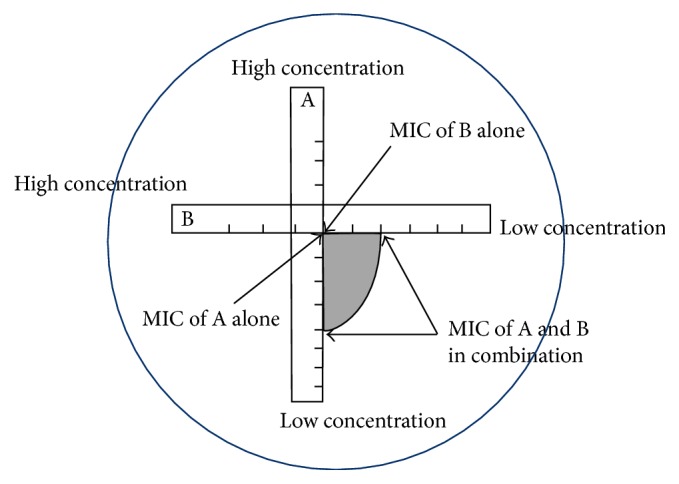
Position of the MIC strips for the antibiotic interaction test: (A) doripenem and (B) amikacin.

The MIC for combined antibiotics was then performed using both strips placed perpendicular and intersect at each MIC as shown in Figure 1. First, the doripenem MIC strip was placed on the MH agar surface, and then, the amikacin MIC strip was put on it at 90° and intersected at each MIC of the isolate against doripenem alone and amikacin alone. Then, both strips were pressed gently, left for 15 minutes, and then incubated for 18–24 h at 35°C. The MIC of each antibiotic and combined antibiotics was read at the tip of inhibitory zone intersected on the E strips [13, 14].

**Figure 2:**
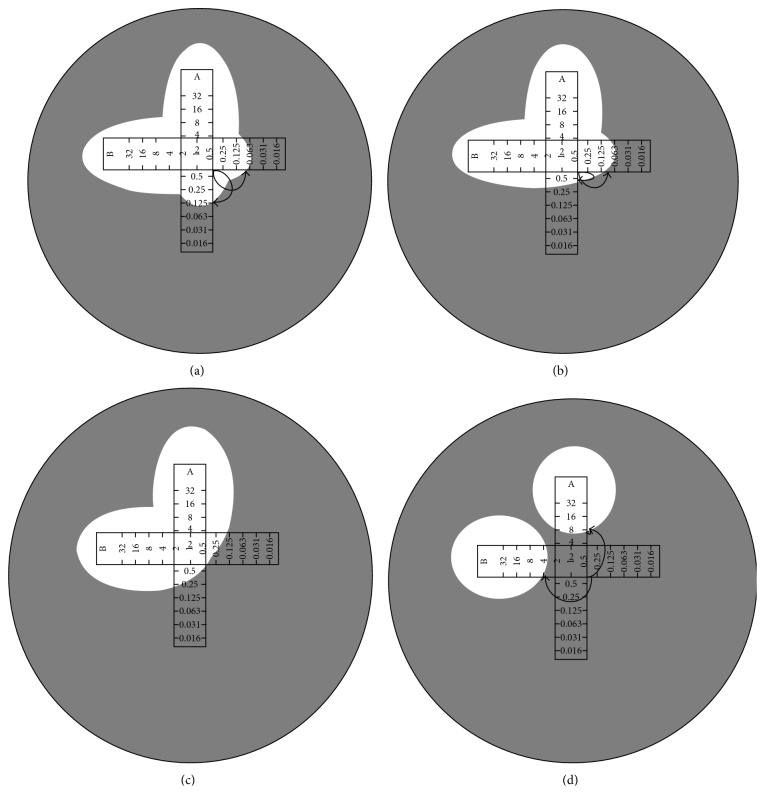
Results of the antibiotic interaction test: (a) synergistic (FIC ≤0.5); (b) additive (FIC >0.5–1.0); (c) indifferent (FIC >1.0–4.0); (d) antagonistic (FIC >4.0) interaction.

To evaluate the effect of the combinations of doripenem (DOR) and amikacin (AMK), the fractional inhibitory concentration (FIC) index was calculated as (MIC_DOR-AMK_/MIC_DOR_) + (MIC_AMK-DOR_/MIC_AMK_). Results were interpreted as synergistic (FIC index ≤0.5), additive (FIC index >0.5–1.0), indifferent (FIC index >1.0–4.0), and antagonistic (FIC index >4.0) interaction as shown in Figure 2 [15].

### 2.4. Statistical Analyses

Results were analyzed and presented descriptively. Mean MIC differences between single and combined antibiotics were analyzed using paired *t*-test or Wilcoxon's signed-rank test. A *p* value of less than 0.05 was considered significant. Statistical analyses were done using the SPSS software version 17.0 (SPSS Inc., Chicago, Illinois, USA).

## 3. Results

During the study period, 80 isolates were collected, but only 68 isolates met the study criteria, consisted of 24 *A. baumannii*, 20 *P. aeruginosa*, and 24 *K. pneumoniae* isolates. There were only 2 isolates of *P. aeruginosa* that were doripenem sensitive and amikacin resistant. Patients' diagnoses varied greatly, that is, 5 (7%) burn wounds, 4 (6%) sepsis, 4 (6%) hospital-acquired pneumonia, 3 (4%) tuberculosis, 2 (3%) chronic kidney disease, 2 (3%) healthcare-associated pneumonia, 2 (3%) postlaparatomy wound, 2 (3%) liver abscess, 2 (3%) ulcers, and many other diagnosis. The most common specimen was sputum (44%), followed by tissue or pus or wound (24%), bronchoalveolar lavage (7%), urine (6%), blood (4%), feces (4%), drain (3%), CVC tip (3%), liver abscess (2%), bronchial fluid (2%), and vitreous fluid (2%). Distribution of each pathogen according to the resistant pattern and source of specimens is given in Table 1.

Sensitivity pattern of the isolates was 5 (7.5%) non-MDR, 24 (35.3%) MDR, 34 (50%) XDR, and 5 (7.5%) PDR. The XDR *K. pneumoniae* has the highest proportion. Within the XDR bacteria group, the XDR carbapenem-resistant isolate was found in 9/24 (37.5%) of *A. baumannii*, 10/20 (50.0%) of *P. aeruginosa*, and 9/24 (37.5%) of *K. pneumoniae* isolates. Only 1 MDR carbapenem-resistant isolate was found in each of the three bacterial isolates.

### 3.1. In Vitro Antibiotic Interaction Test

**Figure 3:**
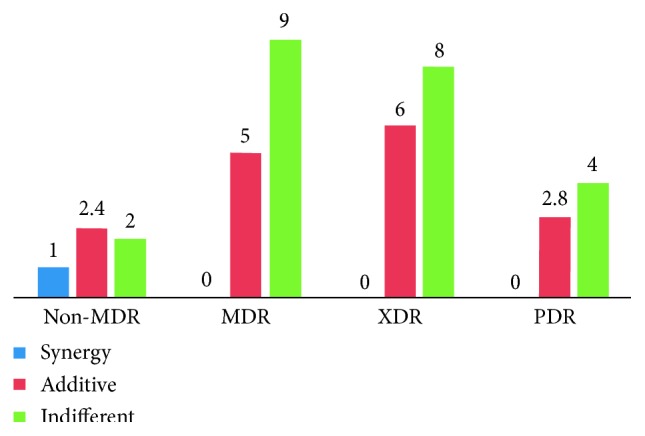
Results of the doripenem-amikacin interaction test based on the drug-resistant class.

Interaction test was done between doripenem and amikacin. The results showed that most isolates were indifferent (63.2%). Except for *P. aeruginosa*, there were 6 isolates allocated in each group of doripenem-amikacin interaction. Only two *P. aeruginosa* isolates were available in the DOR-S/AMK-R group. Synergistic interaction occurred in only 1 (1.5%) isolate, that is, *A. baumannii* within the DOR-S/AMK-S group. The most common result was the indifferent interaction, which occurred in 43 (63.2%) isolates. Additive interaction occurred in 24 (35.3%) isolates. No antagonistic interaction was found (Table 2). Based on drug-resistant class, synergy was only found in 1 non-MDR isolate. Additive interaction was found almost similar between MDR and XDR isolates, but lower in PDR group (Figure 3).

Significant MIC reduction was observed in DOR-S/AMK-S in all bacterial isolates compared to single MIC of each antibiotic. However, most of the significant amount of MIC reduction was not enough to reach synergistic effect and could only reach additive or indifferent effect. On the contrary, combination of doripenem-resistant and amikacin-resistant isolates did not change the MIC. Significant MIC reduction was shown by *K. pneumoniae* isolates when combining either doripenem- or amikacin-resistant isolates with the sensitive counterpart (Table 3).

## 4. Discussion

The antibiotic interaction test using MIC strips is a relatively easy procedure to find the best combination of available antibiotics against MDR pathogens. This study is the first to look for synergy between doripenem and amikacin against the 3 most common resistant Gram-negative bacteria in Cipto Mangunkusumo National Central General Hospital, Jakarta. In this study, specimens were obtained from all wards or polyclinics in the hospital, which were taken from various kinds of underlying diseases. Some studies had used resistant isolates from the intensive care unit only [16, 17], while others used specimens from hospital surveillance without further detail [15, 18].

Our study showed that despite high resistance to doripenem and amikacin, some interactions did occur if both agents were combined resulting in lower MICs than the single antibiotic alone. Lower MIC means the combined antibiotics become more powerful to kill the bacteria. This was more obvious with doripenem sensitive rather than amikacin sensitive. However, antibiotics synergy was only found in one isolate with non-MDR pathogen which showed a synergy with combined doripenem sensitive and amikacin sensitive.

Other study using the checkerboard method showed synergy in 4 of 22 (18.2%) doripenem-resistant *A. baumannii* isolates when combined with amikacin. Further test conducted by another study using the time-kill curve method showed that only 1 of 8 isolates showed synergy while the rest showed indifferent interaction [16]. Previously, a study using the time-kill curve method on 25 isolates of MDR *A. baumannii* showed maximum synergistic effect at 24 hours of incubation period, among which 24 (96%) isolates showed synergistic interaction [18]. In addition, lower synergy interaction has been reported among XDR *A. baumannii* (4% of 48 isolates) [17].

Synergistic interaction in doripenem sensitive and amikacin sensitive can be due to the doripenem effect in destroying the bacterial cell wall that facilitates amikacin influx. Reduced synergy can be due to enzyme inactivation of the antibiotic, such as beta-lactamase (e.g., carbapenemase) or aminoglycoside-modifying enzyme (AME) produced by the bacteria, modification of antibiotic target, decrease in outer membrane permeability, or efflux pump activation [19].

Combined doripenem and amikacin showed different results against *P. aeruginosa*. A study among 100 isolates of carbapenem-resistant *P. aeruginosa* (89 doripenem resistant and 30 amikacin resistant) found 20% synergistic, 47% additive, and 33% indifferent interactions by using the Etest method [15]. No synergistic effect was found when the bacteria produced metallo-*β*-lactamase. Another study using the time-kill curve method on 25 *P. aeruginosa* isolates (4 isolates were doripenem resistant) showed synergy in 22 (88%) isolates treated with combined doripenem and amikacin [18].

In the current study, interaction of doripenem-sensitive isolates was similar with doripenem resistant if the pathogen was also resistant to amikacin. Better interaction was showed by amikacin-sensitive *P. aeruginosa* in doripenem-sensitive compared to doripenem-resistant isolates (Table 3). Additionally, the MIC of doripenem-sensitive *P. aeruginosa* was much higher in the amikacin-resistant group when compared to the amikacin-sensitive group (Table 3). This could be due to the cumulative effects of nonenzymatic mechanism of resistance, which develop gradually after several treatments with aminoglycosides [20].

Interaction tests on *K. pneumoniae* showed varying results. A study using broth microdilution against XDR *K. pneumoniae* isolates from patients with hospital-associated infections (all were doripenem resistant) showed synergy in 10% isolates when combined with amikacin. Doripenem can reduce the amikacin MIC 4–32 times lower and amikacin can decrease doripenem MIC 8–16 times lower to reach sensitive breakpoints [17]. Synergistic effect was also observed when amikacin was combined with meropenem or imipenem against *Klebsiella pneumoniae* carbapenemase (KPC)-producing *K. pneumoniae* [21].

Overall, our study showed that the antibiotic interaction depends on the resistance pattern of the pathogen tested. Combined antibiotics did lower the MIC to some level although synergy can only be achieved when both agents are still sensitive. Higher resistance pattern results in lower interaction of combined antibiotics. However, in this study, we did not check the mechanism of resistance and thus may limit further assessment on its association with the interaction test results.

## 5. Conclusion

In vitro antibiotic interaction test is a useful method to know whether combination of two different antibiotics will be effective to kill multidrug-resistant bacteria. Combination of doripenem and amikacin against *Acinetobacter baumannii*, *Pseudomonas aeruginosa*, and *Klebsiella pneumoniae* showed different interaction effect, which depends on their resistant pattern. Synergistic effect may be difficult to reach, but additive interaction can still be obtained with doripenem sensitive/amikacin resistant or doripenem resistant/amikacin sensitive. When both agents are resistant, nearly all tests showed indifferent interaction. When combined with amikacin, doripenem has better killing effect on *Acinetobacter baumannii* and *Pseudomonas aeruginosa* (nonfermenter Gram-negative bacteria) when compared with *Klebsiella pneumoniae* (fermenter Gram-negative bacteria).

## Figures and Tables

**Table 1:** Distribution of each pathogen based on the drug-resistant patterns and sources (*n*=68).

Source	Non-MDR	MDR	XDR	PDR
	*n* (%)	*n* (%)	*n* (%)	*n* (%)
*A. baumannii*				
Sputum (*n*=15)	1 (50.0)	6 (66.7)	7 (63.6)	1 (50.0)
Tissue (*n*=3)	—	1 (11.1)	2 (18.2)	—
Blood (*n*=2)	—	1 (11.1)	1 (9.1)	—
BAL (*n*=2)	1 (50.0)	—	—	1 (50.0)
Urine (*n*=1)	—	—	1 (9.1)	—
Stool (*n*=1)	—	1 (11.1)	—	—

*P. aeruginosa*				
Sputum (*n*=7)	—	5 (55.6)	2 (20.0)	—
Tissue (*n*=2)	—	—	1 (10.0)	1 (100)
BAL (*n*=1)	—	—	1 (10.0)	—
Urine (*n*=1)	—	1 (11.1)	—	—
Wound (*n*=3)	—	—	3 (30.0)	—
Pus (*n*=1)	—	—	1 (10.0)	—
Others (*n*=5)	—	3 (33.3)	2 (20.0)	—

*K. pneumoniae*				
Sputum (*n*=8)	2 (66.7)	2 (33.3)	3 (23.1)	1 (50.0)
Tissue (*n*=1)	—	—	1 (7.7)	—
Blood (*n*=1)	—	—	1 (7.7)	—
BAL (*n*=2)	—	1 (16.7)	1 (7.7)	—
Urine (*n*=2)	1 (33.3)	—	1 (7.7)	—
Stool (*n*=2)	—	2 (33.3)	—	—
Wound (*n*=2)	—	—	1 (7.7)	1 (50.0)
Pus (*n*=2)	—	—	2 (15.4)	—
Others (*n*=4)	—	1 (16.7)	3 (23.1)	—

BAL: bronchoalveolar lavage.

**Table 2:** Results of doripenem and amikacin interaction test.

Classification	*A. baumannii*	*P. aeruginosa*	*K. pneumoniae*	Total
*DOR-S/AMK-S (n*=18)				
Synergy	1	0	0	1
Additive	5	5	2	12
Indifferent	0	1	4	5
Antagonist	0	0	0	0

*DOR-S/AMK-R (n=14)*				
Synergy	0	0	0	0
Additive	3	0	1	4
Indifferent	3	2	5	10
Antagonist	0	0	0	0

*DOR-R/AMK-S (n=18)*				
Synergy	0	0	0	0
Additive	1	1	4	6
Indifferent	5	5	2	12
Antagonist	0	0	0	0

*DOR-R/AMK-R (n=18)*				
Synergy	0	0	0	0
Additive	0	1	1	2
Indifferent	6	5	5	16
Antagonist	0	0	0	0

**Table 3:** Mean MIC changes after combining doripenem and amikacin.

Bacteria	MIC_DOR_	MIC_DOR+AMK_	Mean difference	*p* value^*∗*^	MIC_AMK_	MIC_AMK+DOR_	Mean difference	*p* value^*∗*^
DOR-S/AMK-S								
*A. baumannii*	0.37	0.13	0.24	**0.001**	2.33	0.88	1.45	**0.008**
*P. aeruginosa*	0.66	0.32	0.34	**0.006**	4.00	1.69	2.31	**0.003**
*K. pneumoniae*	0.29	0.09	0.20	**0.027^**	3.25	2.42	0.83	**0.041^**

DOR-S/AMK-R								
*A. baumannii*	4.08	2.33	1.75	0.073	121.33	92.67	28.67	0.221
*P. aeruginosa*	4.00	3.00	1.00	0.157^	256.00	192.00	64.00	0.157
*K. pneumoniae*	0.09	0.06	0.03	**0.033**	217.33	162.67	54.66	**0.035**

DOR-R/AMK-S								
*A. baumannii*	32.00	29.33	2.67	0.363	3.58	2.75	0.83	0.129
*P. aeruginosa*	32.00	27.67	4.33	0.363	9.00	7.67	1.33	0.363
*K. pneumoniae*	26.67	9.12	17.50	**0.001**	2.50	1.50	1.00	**0.018**

DOR-R/AMK-R								
*A. baumannii*	32.00	32.00	0.00	0.317^	256.00	256.00	0.00	0.109^
*P. aeruginosa*	32.00	27.33	4.67	0.180	141.33	128.00	13.33	0.180
*K. pneumoniae*	23.33	21.33	2.00	0.203	256.00	220.33	26.67	0.363

^*∗*^Paired *t*-test; ^Wilcoxon's signed-rank test.

## Data Availability

The data used to support the findings of this study are available from the corresponding author upon request.
